# A mixed-methods study on the association of six-month predominant breastfeeding with socioecological factors and COVID-19 among experienced breastfeeding women in Hong Kong

**DOI:** 10.1186/s13006-022-00484-7

**Published:** 2022-05-21

**Authors:** John Kwan, Jimsyn Jia, Ka-man Yip, Hung-kwan So, Sophie S. F. Leung, Patrick Ip, Wilfred H. S. Wong

**Affiliations:** 1 Department of Paediatrics and Adolescent Medicine, The University of Hong Kong, Hong Kong SAR, China; 2 Department of Paediatrics, The Chinese University of Hong Kong, Hong Kong SAR, China

**Keywords:** Breastfeeding, Predominant breastfeeding, COVID-19, Family support, Public hospital, Private hospital, Baby-friendly hospital initiative, Antenatal class, Knowledge, Hong Kong

## Abstract

**Background:**

In the past decade, various breastfeeding policies were implemented in Hong Kong, including changes in perinatal guidelines in public hospitals, adoption of the Baby-Friendly Hospital Initiative (BFHI), provision of guidelines for the marketing of formula milk, penalisation of discrimination towards breastfeeding, and extension of the statutory maternity leave. Meanwhile, the COVID-19 pandemic brought new challenges and opportunities to breastfeeding practices. Infection control measures in public hospitals included the cancellation of antenatal classes, hospital tours, and postnatal classes; suspension of perinatal visiting periods; and compulsory separation of COVID-19 positive mothers from newborns. In addition, work-from-home policies were widely implemented. This study aimed to identify the associated factors of six-month predominant breastfeeding (PBF), and to evaluate the impact of COVID-19 on breastfeeding practice.

**Methods:**

This study was conducted from 1 March 2021 to 7 April 2021 using a mixed-methods approach. An electronic questionnaire was distributed to members of breastfeeding or parenting groups who have had breastfeeding experience in the past 10 yrs. Logistic and linear regression analyses were conducted to identify factors associated with six-month PBF both in general and during the pandemic period. A qualitative content analysis was conducted using an inductive approach.

**Results:**

The study included 793 participants. Giving birth in a public hospital (OR 2.21; 95% CI 1.46, 3.34) and breastfeeding support from family and friends (OR 1.28; 95% CI 1.05, 1.57) were significantly associated with six-month PBF, even during COVID-19. Factors associated with the self-rated impact of COVID-19 on breastfeeding include working from home, the perceived immunological benefits of breastfeeding, and the wish to avoid breastfeeding or expressing breast milk in public premises. Furthermore, breastfeeding practice in public hospitals was more likely to be affected by the busyness of staff, while private hospitals had worse rooming-in practices and staff who had inadequate breastfeeding knowledge.

**Conclusions:**

Giving birth in a public hospital and having breastfeeding support from family and friends were associated with six-month PBF. Furthermore, COVID-19 in Hong Kong had an overall positive impact on six-month PBF. Further studies should investigate the impact of hospital practices and the COVID-19 pandemic on breastfeeding behaviours.

**Supplementary Information:**

The online version contains supplementary material available at 10.1186/s13006-022-00484-7.

## Background

In Hong Kong, various policies have been developed to support breastfeeding over the past decade. In 2010, public hospitals in Hong Kong stopped accepting free breast milk substitutes from manufacturers [1]; such a pivotal policy reduced the availability of formula and encouraged breastfeeding practices amongst new breastfeeding mothers. Meanwhile, the Hospital Authority is committed to supporting all eight public hospitals with obstetric wards to comply with the World Health Organization (WHO)-launched Baby-Friendly Hospital Initiative (BFHI) and implement the “Ten Steps to Successful Breastfeeding”. In 2017, the Food and Health Bureau issued the Hong Kong Code of Marketing of Formula Milk and Related Products, and Food Products for Infants & Young Children to protect breastfeeding in response to the aggressive marketing of formula milk in Hong Kong [2]. Recently, the Discrimination Legislation (Miscellaneous Amendments) Ordinance 2020 came into force to protect breastfeeding women from direct or indirect discrimination, and the Employment (Amendment) Ordinance 2020 was established to extend statutory maternity leave from 10 weeks to 14 weeks [3, 4].

Consequently, the breastfeeding initiation rate of Hong Kong increased significantly from 76.7% (2010) to 87.2% (2019), keeping up with other Asian jurisdictions such as the Republic of Korea (90.2%) [5, 6]. Despite this, the exclusive breastfeeding (EBF) rate at the first month remained low at 32.6% in Hong Kong, while the EBF rate at the sixth month further dropped to 26.3% [7], outperforming the Republic of Korea (2.3%), but underperforming Japan (37.4%) and Taiwan (46.2%) [6–9]. Moreover, early breastfeeding cessation remains common in Hong Kong. Between the first and sixth months, 19.3% of EBF mothers discontinued EBF [7]. To support sustainable breastfeeding practices and raise the breastfeeding rate, it is important to identify barriers to breastfeeding. Well-established barriers include poor family and workplace support, lack of knowledge about the superior health benefits of breastfeeding, inadequate breastfeeding skills, and sociocultural norms and beliefs [10].

During the COVID-19 pandemic which first struck Hong Kong in January 2020, the government and different organisations implemented work-from-home arrangements, while breastfeeding coaching services in maternal and child health centres (MCHCs), hospital tours, and postnatal classes were temporarily suspended or cancelled [11–13]. In public hospitals, perinatal visiting periods were suspended, while COVID-19 − positive mothers were compulsorily separated from their newborns to prevent transmission, although bottle-feeding of expressed breast milk was allowed [14]. Although it is believed that the breastfeeding rate would be affected by these measures, limited studies have been conducted to examine the impact of COVID-19 on breastfeeding internationally, while none were conducted in Hong Kong [15].

Predominant breastfeeding (PBF) is defined as the predominant consumption of breast milk, with potential consumption of water or water-based drinks (sweetened or flavoured water, teas, infusions, etc.), fruit juice, oral rehydration salts, drop and syrup forms of vitamins, minerals, and medicines, and folk fluids (in limited quantities) [16]. This study aimed to identify socioecological factors associated with the practice of PBF for at least 6 months since birth (hereafter referred to as “six-month PBF”), with a focus on maternal breastfeeding support, antenatal education, intrapartum breastfeeding assistance offered by hospitals, and postpartum education. The study also explored the perceptions of Hong Kong women who have had experience breastfeeding within the past 10 years. Furthermore, this study aimed to evaluate the impact of COVID-19 on PBF and investigate the unique opportunities and challenges for breastfeeding brought by COVID-19. In addition, this paper will provide recommendations for encouraging PBF.

## Methods

### Study design and participants

This study was conducted using a quantitatively driven mixed-methods research design comprising both quantitative and qualitative analytical approaches, in which qualitative data were collected to supplement or interpret the quantitative results. Both quantitative and qualitative data were collected via an electronic questionnaire. A purposive sampling approach was adopted to target mothers with breastfeeding experience, breastfeeding determination, and experience with a variety of breastfeeding services provided by various health care providers, organisations, and professionals. Hence, only members of breastfeeding or parenting groups were invited to participate in the study; such support groups include the Hong Kong Breastfeeding Mothers’ Association (HKBFMA) and La Leche League Hong Kong (LLLHK). Specifically, the inclusion criteria were Hong Kong mothers with breastfeeding experience in the past 10 yrs, so as to evaluate the multitude of breastfeeding policies that were put forth since 2010.

### Instrument

An electronic questionnaire was developed to investigate the breastfeeding practices and experiences of Hong Kong mothers (see Additional file 1). Questionnaire items were selected based on previous published investigations in Hong Kong or their relevance to the objectives of this study [17–19]. The questionnaire explores five areas of interest, namely (a) participants’ perceived breastfeeding support from their family and friends, workplace, and public premises, (b) participants’ perceived helpfulness of antenatal classes, (c) participants’ experience in hospital maternity wards during the perinatal period, (d) participants’ postpartum experience with different health organisations and professionals, including MCHCs, breastfeeding support organisations, breastfeeding clinics, International Board Certified Lactation Consultants (IBCLCs), private doctors, private nurses, confinement nannies, and milk enhancing workers, and (e) participants’ perceived impact of COVID-19 on breastfeeding. Question types included Likert scale, polar, categorical, and open-ended questions.

In particular, breastfeeding support from family and friends was measured using three questions from Section 4 of the questionnaire: (1) “My spouse is supportive of me breastfeeding my child”, (2) “My parents are supportive of me breastfeeding my child”, and (3) “My friends are supportive of me breastfeeding my child”. Likert scale format was used for participants’ response options: from 1 (strongly disagree) to 5 (strongly agree). The internal consistency of this variable was assessed by Cronbach’s alpha (value = 0.681).

An independent panel of six members, consisting of registered nurses, IBCLCs, and paediatricians, was consulted during the questionnaire development process. The survey was piloted with the panel to assess the validity of the questionnaire.

### Data collection

The questionnaire was distributed on the Facebook platform of HKBFMA and LLLHK. Responses were collected from 1 March 2021 to 7 April 2021. The questionnaire took less than 15 minutes to be completed, and study results would be shared with members of HKBFMA and LLLHK in order to motivate participants and increase the response rate. Prior to answering the questionnaire, participants were assured about the confidentiality and anonymity of the data collected and understood that they could withdraw from the study at any time. To ensure the quality of data collected, algorithms were employed in the electronic questionnaire to direct the sequence of questions, ensure no contradicting responses, and mandate answering of close-ended questions.

## Analysis

### Statistical analysis

Descriptive statistics were used to examine the characteristics of all participants. Examined characteristics include maternal age, maternal education level, maternal employment status, monthly total household income, the size of residential property, number of people in the household, and birth hospital (public or private). Principal component analysis was used to create a socioeconomic status (SES) index with household income per capita, maternal age, maternal education level, and maternal employment status. For participants whose child was 6 months or older, chi-square test and Fisher’s exact test were used to compare their categorical variables in relation to their practice of six-month PBF, which was calculated based on the onset and duration of breastfeeding, formula milk feeding, and solid food intake, answered in Section 2 of the questionnaire.

Descriptive statistics were also used to examine participants’ (I) reasons for EBF cessation, (II) perceived helpfulness of antenatal classes, (III) experience in hospital maternity wards during the perinatal period, and (IV) postpartum experience with different health organisations and professionals. Chi-square tests were further performed for part (III) to compare the breastfeeding difficulties encountered by participants in public and private hospitals.

To determine factors associated with successful six-month PBF in participants whose child was 6 months or older, univariable binary logistic regression analysis was performed for each of the following independent variables: birth hospital (public or private), attendance of antenatal classes, seeking of lactation consultation due to breastfeeding difficulties after birth, and breastfeeding support from family and friends. Multiple logistic regression analysis was then performed with significant variables, adjusted for the SES index.

To investigate the impact of the COVID-19 pandemic on successful six-month PBF, univariable binary logistic regression analysis was performed among participants whose child was six to 12 months old for each of the following independent variables: birth hospital (public or private), attendance of antenatal classes, seeking lactation consultation due to breastfeeding difficulties after birth, breastfeeding support from family and friends, and self-rated impact of COVID-19 on breastfeeding. Multiple logistic regression analysis was then performed with significant variables, adjusted for the SES index.

To investigate the reasons behind self-rated impact of COVID-19 on breastfeeding (listed in Section 8 of the questionnaire), collinearity of the independent variables was first assessed using the Pearson correlation test, after which univariable linear regression analysis was performed. Multiple linear regression analysis was subsequently performed on the significant variables.

Two-sided *p*-value of less than 0.05 was considered statistically significant. All analyses were performed using IBM SPSS Statistics for Macintosh, version 26.0 (IBM Corp., Armonk, NY, USA).

To ensure the quality of data used, the data of participants with incomplete questionnaire responses were excluded from the analysis. Data cleaning was also conducted to remove illogical responses.

### Qualitative analysis

Qualitative content analysis was performed for all participants. Open-ended responses in the questionnaire were coded using an inductive approach to supplement the quantitative results regarding parts (I) to (IV).

As inspired by Elo and Kyngas, our inductive method consisted of three stages: open coding, categorisation, and abstraction [20]. During the first stage, open-ended responses were read through by the authors to establish the general viewpoint and attitude of participants. Relevant headings were then extracted to generate coding units. During the second stage, similar or related coding units were grouped into higher order categories to reduce the number of codes. During the final stage, themes were generated based on higher order categories, with reference to characteristic words or syntax, so as to represent the original ideas of participants as closely as possible. The frequency counts of induced themes were then presented for discussion.

Responses regarding participants’ experience in public and private hospital maternity wards and breastfeeding clinics were separately analysed, to contrast the breastfeeding support offered by public and private hospitals.

## Results

A total of 880 responses were made, of which 793 (90.1%) were complete and met the inclusion criteria. Of the 656 participants whose child was 6 months or older, 286 (43.6%) had practised six-month PBF, while 370 (56.4%) had not.

### Characteristics and breastfeeding pattern of participants

Table 1 shows the characteristics and breastfeeding pattern of all participants. The majority (77.4%) of participants were between 31 and 40 years of age, and 82.1% had degrees. There were 57.4% participants employed full time, 9.2% employed part time, 6.9% on maternity leave, and 26.5% were housewives. Amongst all participants, 60.2% gave birth in a public hospital. There were more four-person households (*p* = 0.008) and a greater proportion of public hospital births (*p* <  0.001) in participants who had practised six-month PBF.

**Table 1:** Characteristics and breastfeeding pattern of participants

Characteristic	Total (***n*** = 793) %	Children aged less than 6 months (***n*** = 137) %	Children aged 6 months or more		
			Six-month PBF (***n*** = 286) %	Not six-month PBF (***n*** = 370) %	***p***-value*
**Maternal age**					0.270
21–30	8.6	12.4	7.0	8.4	
31–40	77.4	83.2	74.5	77.6	
≥ 41	14.0	4.4	18.5	14.1	
**Maternal education**					0.757
Secondary or below	7.7	6.6	8.0	7.8	
Post-secondary: diploma / certificate	8.3	13.9	8.4	6.2	
Post-secondary: sub-degree course	1.9	0.7	2.1	2.2	
Post-secondary: degree course	82.1	78.8	81.5	83.8	
**Maternal employment status**					0.638
Employed (full time)	57.4	36.5	59.8	63.2	
Employed (part time)	9.2	4.4	10.1	10.3	
Unemployed	26.5	30.7	26.9	24.6	
On maternity leave	6.9	28.5	3.1	1.9	
**Monthly total household income (HKD)**					0.645
≤ 29,999	7.8	9.8	6.8	7.8	
30,000–49,999	14.4	15.0	16.1	12.7	
50,000–69,999	19.8	23.3	16.8	20.8	
70,000–89,999	15.5	12.0	15.8	16.6	
90,000–109,999	15.0	15.8	16.1	13.9	
≥ 110,000	27.6	24.1	28.3	28.3	
**Size of residential property (sq. ft.)**					0.091
≤ 400	17.3	16.3	16.3	18.5	
400–599	23.4	29.6	18.4	25.0	
600–799	24.4	22.2	25.4	24.5	
≥ 800	34.9	31.9	39.9	32.1	
**Number of people in the household**					0.008
2	0.5	1.5	0.0	0.5	
3	34.6	34.6	28.3	39.6	
4	39.5	35.3	45.2	36.6	
≥ 5	25.4	28.7	26.5	23.3	
**Birth hospital**					< 0.001
Public hospital	60.2	53.3	69.9	55.1	
Private hospital	39.8	46.7	30.1	44.9	

*PBF* predominant breastfeeding

*Six-month PBF versus not six-month PBF

### Reasons for EBF cessation

There were 391 participants who gave reasons for discontinuing EBF. Natural weaning (47.1%), perceived insufficient milk supply (39.4%), life’s busyness (24.0%), and returning to work after maternity leave were all common reasons (21.7%).

In addition, 23 valid open-ended responses were obtained, from which the theme of “maternal mental distress” was induced (*n* = 9).

### Perceived helpfulness of antenatal classes

Of the 577 participants who had attended antenatal classes, 37.4% found them unhelpful in guiding breastfeeding, 36.7% found them helpful, and 25.8% remained neutral.

In addition, 317 valid open-ended responses were collected. Induced themes are summarised in Table 2.

**Table 2:** Induced themes regarding antenatal classes

Themes	Categories	Frequency (n)
Ineffective nature of antenatal teaching	Breastfeeding could only be effectively learnt postpartum	92
	Breastfeeding could only be effectively learnt practically	
	Breastfeeding cannot be effectively learnt during the antenatal period	
Inadequate information	Information was incomprehensive	75
	Information was generic	
	Advice and teaching were theoretical	
	Breastfeeding challenges were understated	
Basic but helpful information	Information was basic but helpful	58
	Information offered mothers a head start	
Lack of personalisation	Antenatal classes were not customised to tackle unique breastfeeding challenges encountered by individual mother-infant pairs	38
Lack of time or emphasis on breastfeeding issues	Insufficient time was allocated to teaching breastfeeding issues	32
	Insufficient emphasis on breastfeeding issues	
Antenatal classes were affected by COVID-19	Classes were cancelled due to COVID-19	4
	Classes were taught online due to COVID-19	

### Experience in hospital maternity wards during the perinatal period

Around 79% of participants who gave birth in public hospitals encountered some form of breastfeeding problems, while nearly 83% of participants who gave birth in private hospitals did so. Difficulties encountered in maternity wards are summarised in Table 3.

**Table 3:** Difficulties encountered in maternity wards

	Public ***N*** = 477 (%)	Private ***N*** = 316 (%)	***p***-value
Latching issues	263 (55.1)	165 (52.2)	0.093
Need to supplement with other milk sources due to low breast milk supply	131 (27.5)	127 (40.2)	< 0.001
Unable to initiate breastfeeding within 30 minutes after delivery / becoming responsive	131 (27.5)	111 (35.1)	0.044
Nurses were too busy to offer effective breastfeeding assistance	138 (28.9)	48 (15.2)	< 0.001
Midwives were too busy to offer effective breastfeeding assistance	97 (20.3)	24 (7.6)	< 0.001
None	99 (20.8)	55 (17.4)	0.243

A problem with latching was the most common breastfeeding problem encountered in both public maternity wards (55.1%) and private maternity wards (52.2%); the prevalence of latching problem in public and private maternity wards showed no significant difference (*p* = 0.093). However, compared to public hospital participants, a significantly greater proportion of private hospital participants supplemented with other milk sources due to perceived insufficient milk supply (40.2% > 27.5%, *p* <  0.001) and failed to initiate breastfeeding within 30 minutes after delivery or being responsive (35.1% > 27.5%, *p* = 0.044). Meanwhile, compared to private hospital participants, a significantly greater proportion of public hospital participants did not receive effective breastfeeding assistance due to the busyness of nurses (28.9% > 15.2%, *p* <  0.001) and midwives (20.3% > 7.6%, *p* <  0.001).

In addition, 60 valid open-ended responses regarding perinatal support from public hospitals were collected, while 21 responses regarding that of private hospitals were collected. Induced themes are summarised in Table 4.

**Table 4:** Induced themes regarding perinatal support from public and private hospitals

Themes	Categories	Frequency (n)
**Public hospitals**		
Inadequate breastfeeding support in SCBUs and NICUs	Visiting, milk expressing, breastfeeding, and bottle-feeding policies were unclear or poorly communicated	17
	Visiting hours and number of visitors were limited	
**Private hospitals**		
Inadequate breastfeeding knowledge of staff	Nurses inappropriately recommended formula feeding as first-line solution for all breastfeeding issues	7
	Midwives or nurses offered conflicting and incomprehensive breastfeeding advice	
Poor rooming-in practice	Infants were unnecessarily separated from mothers due to non-medical reasons	3

*NICU* neonatal intensive care units, *SCBU* special care baby unit

### Postpartum experience with different health organisations and professionals

Of all participants who sought breastfeeding coaching services due to breastfeeding difficulties (*n* = 503), most sought help from MCHCs (58.3%), IBCLCs (32.4%), breastfeeding support organisations (29.2%), and confinement nannies (23.3%).

Open-ended responses indicated that IBCLCs, private nurses, and certified milk enhancing workers were available for home visits and “personalised support”, while private breastfeeding clinics, private doctors, and confinement nannies received mixed open-ended responses regarding their “supportiveness”, “breastfeeding knowledge”, and “experience with breastfeeding”. In addition, 15 participants were unaware of the postnatal service provided by MCHCs.

### Factors associated with successful six-month PBF

The binary logistic regression models are shown in Tables 5 and 6. Univariable regression analysis indicated that the type of birth hospital, whether lactation consultation was sought due to breastfeeding difficulties after birth, and breastfeeding support from family and friends were associated with successful six-month PBF (Table 5). Multiple logistic regression showed that giving birth in a public hospital (OR 2.21; 95% CI 1.46, 3.34) and breastfeeding support from family and friends (OR 1.28; 95% CI 1.05, 1.57) were associated with successful six-month PBF (Table 6).

**Table 5:** Results of univariable logistic regression analysis on successful six-month PBF

Factor	β	SE	OR	***P***-value	95% CI for OR
Birth hospital					
Public	0.64	0.17	1.89	< 0.001	1.37 to 2.62
Private (reference)					
Attended antenatal classes					
Yes	−0.09	0.18	0.91	0.607	0.64 to 1.30
No (reference)					
Sought lactation consultation due to breastfeeding difficulties					
Yes	−0.55	0.17	0.58	0.001	0.42 to 0.80
No (reference)					
Breastfeeding support from family and friends	0.28	0.09	1.32	0.001	1.12 to 1.56

*CI* confidence interval, *OR* odds ratio, *SE* standard error

**Table 6:** Results of multiple logistic regression analysis on successful six-month PBF

Factor	β	SE	OR	***P***-value	95% CI for OR
Birth hospital					
Public	0.79	0.21	2.21	< 0.001	1.46, 3.34
Private (reference)					
Sought lactation consultation due to breastfeeding difficulties					
Yes	−0.32	0.20	0.72	0.106	0.49, 1.07
No (reference)					
Breastfeeding support from family and friends	0.25	0.10	1.28	0.015	1.05,1.57

*CI* confidence interval, *OR* odds ratio, *SE* standard error* Model was adjusted with the SES index

### Factors associated with successful six-month PBF during COVID-19

The binary logistic regression models are shown in Tables 7 and 8. Univariable regression analysis indicated that the type of birth hospital, breastfeeding support from family and friends, and self-rated impact of COVID-19 on breastfeeding were associated with successful six-month PBF during COVID-19 (Table 7). Multiple logistic regression showed that giving birth in a public hospital (OR 3.57; 95% CI 1.84, 6.90), breastfeeding support from family and friends (OR 1.43; 95% CI 1.00, 2.02), and self-rated impact of COVID-19 on breastfeeding (OR 1.21; 95% CI 1.07, 1.38) were associated with successful six-month PBF during COVID-19 (Table 8).

**Table 7:** Results of univariable logistic regression analysis on successful six-month PBF during COVID-19

Factor	β	SE	OR	***p***-value	95% CI for OR
Birth hospital					
Public	0.94	0.25	2.56	< 0.001	1.58, 4.16
Private (reference)					
Attended antenatal classes					
Yes	0.01	0.27	1.01	0.959	0.60,1.71
No (reference)					
Sought lactation consultation due to breastfeeding difficulties					
Yes	−0.41	0.25	0.67	0.105	0.41,1.09
No (reference)					
Breastfeeding support from family and friends	0.31	0.14	1.36	0.024	1.04,1.77
Self-rated impact of COVID-19 on breastfeeding	0.10	0.05	1.11	0.038	1.01,1.22

*CI* confidence interval, *OR* odds ratio, *SE* standard error

**Table 8:** Results of multiple logistic regression analysis on successful six-month PBF during COVID-19

Factor	β	SE	OR	***p***-value	95% CI for OR
Birth hospital					
Public	1.27	0.34	3.57	< 0.001	1.84, 6.90
Private (reference)					
Breastfeeding support from family and friends	0.35	0.18	1.43	0.047	1.00, 2.02
Self-rated impact of COVID-19 on breastfeeding	0.19	0.07	1.21	0.004	1.07,1.38

*CI* confidence interval, *OR* odds ratio, *SE* standard error* Model was adjusted with the SES index

Of the 296 participants whose child was six to 12 months old, 53% rated the overall impact of COVID-19 on breastfeeding as positive, while 41.2% rated it as neutral. The linear regression models on the reasons for self-rated impact of COVID-19 on breastfeeding are shown in Tables 9 and 10. Being able to work from home and the perceived advantage of breastfeeding in improving children’s immunity were reasons that encouraged breastfeeding during the pandemic, whereas avoiding breastfeeding or expressing breast milk in public premises to reduce children’s risk of infection was a significant reason that discouraged breastfeeding (Table 10).

**Table 9:** Results of univariable linear regression analysis on reasons for self-rated impact of COVID-19 on breastfeeding

Factor	β	S.E.	95% CI for β	T-value	***p***-value
I can work from home.	1.33	0.27	0.8, 1.87	4.90	< 0.001
I leave home less during the pandemic.	0.62	0.31	0.02, 1.22	2.02	0.044
My family members can better support breastfeeding at home.	0.69	0.34	0.01, 1.37	2.01	0.045
Breastfeeding can improve infants’ immunity.	1.19	0.30	0.60, 1.77	3.97	< 0.001
It is easier to seek help in regards to breastfeeding.	0.44	0.51	−0.56, 1.44	0.87	0.386
It is more difficult to seek help in regards to breastfeeding.	−2.04	1.00	−4.00, −0.08	−2.05	0.042
I want to minimise physical contact with my children, so as to reduce their chance of being infected.	−0.14	0.93	−1.98, 1.69	−0.15	0.878
I want to avoid breastfeeding / expressing breast milk in public, so as to reduce my children’s chance of being infected.	−1.19	0.40	−1.98, −0.39	−2.94	0.004

*CI* confidence interval, *OR* odds ratio, *SE* standard error

**Table 10:** Results of multiple linear regression analysis on reasons for self-rated impact of COVID-19 on breastfeeding

Factor	β	S.E.	95% CI for β	T-value	***p***-value
I can work from home.	1.25	0.27	0.72, 1.78	4.65	< 0.001
I leave home less during the pandemic.	0.25	0.30	−0.34, 0.85	0.83	0.407
My family members can better support breastfeeding at home.	0.29	0.34	−0.37, 0.96	0.87	0.387
Breastfeeding can improve infants’ immunity.	1.05	0.30	0.47, 1.63	3.55	< 0.001
It is more difficult to seek help in regards to breastfeeding.	−1.02	0.93	−2.86, 0.82	−1.09	0.277
I want to avoid breastfeeding / expressing breast milk in public, so as to reduce my children’s chance of being infected.	−1.52	0.39	−2.28, −0.76	−3.92	< 0.001

## Discussion

This study complemented the existing literature regarding the barriers and facilitators of breastfeeding by identifying three factors associated with successful six-month PBF, namely giving birth in a public hospital, the self-rated impact of COVID-19, and the support from family and friends. Such factors could be targeted by policymakers for designing breastfeeding interventions.

### Breastfeeding assistance offered by hospitals

Both descriptive statistics and open-ended responses regarding the perinatal experience of participants indicated a high prevalence of breastfeeding problems in both public and private maternity wards, suggesting that neonatal support for breastfeeding in both public and private hospitals was generally insufficient and ineffective. In particular, giving birth in private hospitals was associated with a lower likelihood of successful six-month PBF, both in general and during the pandemic period. There are four potential reasons why private hospitals discourage EBF. First, the inappropriate recommendation of formula supplementation by staff in private hospitals likely reinforced perceived insufficient milk supply amongst mothers, hence discouraging them from in-hospital EBF (BFHI Step 6) and responsive breastfeeding (BFHI Step 8) [21]. As demonstrated in both our study and other international studies, perceived insufficient milk supply is a major factor for discontinued breastfeeding and EBF cessation [22–24]. Moreover, while in-hospital EBF has been demonstrated to be positively associated with continued breastfeeding or EBF in both local and international studies, in-hospital formula supplementation is a risk factor for early breastfeeding cessation worldwide [25–28]. Second, compared to public hospitals, private hospitals were less supportive of early in-hospital breastfeeding initiation (BFHI Step 4), a finding consistently implied from both local and international studies [5, 21, 29]. Yet, early in-hospital breastfeeding initiation is a factor strongly positively associated with breastfeeding establishment, in-hospital EBF, and continued breastfeeding worldwide [21, 28, 30]. Third, private hospital staff tended to offer conflicting and incomprehensive breastfeeding advice to mothers. This is likely due to the relatively poor communication of hospital breastfeeding policies (BFHI Step 1) and insufficient staff training (BFHI Step 2) in private hospitals as compared to public hospitals [5, 21]. Fourth, private hospitals were generally unsupportive of rooming-in practice (BFHI Step 7), a factor significantly positively associated with in-hospital EBF [21, 31]. The poor rooming-in practice of private hospitals, as compared to public hospitals which all practised rooming-in, was observed in another local survey [5]. Overall, our results suggested that private hospitals were less supportive of in-hospital EBF and less committed to the BFHI than public hospitals, although public hospitals had a shortage of staff and lacked breastfeeding support in special care baby units (SCBUs) and neonatal intensive care units (NICUs). Further investigation is needed to assess whether similar trends are observed internationally.

### Impact of COVID-19

We also found the pandemic in Hong Kong to have a generally positive self-rated impact on breastfeeding, which was positively associated with successful six-month PBF. Our linear regression results suggested that this is likely due to the work-from-home arrangements implemented and the perceived benefits of breastfeeding in improving children’s immunity. In contrast, a study in Italy found lockdown and home confinement measures during COVID-19 to reduce EBF [15]. Such contrasting findings were likely due to two reasons. First, the more severe COVID-19 situation in Italy and uncertainties around vertical transmission of SARS-CoV-2 during the study period (March—August 2020) at the early stages of the pandemic made mothers and healthcare workers alike wary of breastfeeding [32, 33]. Second, due to stringent lockdown restrictions in Italy that resulted in complete home confinement and reduced transportation, new families could not access breastfeeding support easily, including those from family and friends [34]. Meanwhile, the pandemic also presented unique difficulties to breastfeeding. Mothers may avoid breastfeeding and expressing breast milk in public premises to reduce children’s risk of infection. Moreover, the temporary suspension of postnatal services in MCHCs during pandemics could deprive mothers of critical breastfeeding support, hence negatively impact breastfeeding practice amongst mothers. Furthermore, during the pandemic, most antenatal classes and breastfeeding coaching services were cancelled, while some were moved online with the implementation of social distancing measures. Some participants further complained that online antenatal classes did not facilitate interactive teaching and effective learning; similar findings were observed in the UK [35].

### Breastfeeding support

Results of logistic regression models further indicated that breastfeeding support from family and friends was positively associated with successful six-month PBF, both in general and during the pandemic period. This is consistent with previous studies that emphasised the importance of the husband’s preference for EBF and having peers who have had breastfeeding experience, as well as the positive impact of paternity leave on breastfeeding duration [36–39]. In Chinese society, it is traditional for the grandparents to assist new mothers during the first month postpartum and as such, the attitude of grandparents strongly influences feeding practices [38]. The notion that EBF is not enough for the baby’s satiety is strong amongst the older generation and reinforces perceived insufficient milk supply in new mothers, a circumstance that often convinces new mothers to supplement with or switch to formula feeding [40, 41].

### Socioeconomic status

Both local and international studies have demonstrated that the exclusivity of breastfeeding and the duration of EBF are positively associated with maternal education level and household income [42, 43]. However, due to our purposive sampling approach, our participants had a higher education level and socioeconomic status compared to the general population, and were more committed to breastfeeding [44]. As such, these associations were not observed in this study.

### Strengths and limitations

Our study adopted a purposive sampling approach and targeted members of breastfeeding support groups and organisations, who were committed to breastfeeding, experienced with breastfeeding, and exposed to a wide range of breastfeeding services provided by different health care providers, organisations, and professionals. This allowed wide-ranging exploration on the barriers and facilitators of breastfeeding to help inform future research efforts. In addition, this is the first study undertaken to explore the impact of COVID-19 on breastfeeding practice in Hong Kong.

However, our study had some limitations. Due to the voluntary nature of this study, it was possible that those who participated had more positive attitudes towards breastfeeding, subjecting it to volunteer response bias. In addition, the purposive sampling approach limited the generalisability of our findings. Moreover, the inductive method adopted in the qualitative content analysis could oversimplify the individual breastfeeding experience of participants. Furthermore, self-reporting responses of participants were subject to recall bias. Lastly, responses regarding participants’ perceived helpfulness of different health organisations and professionals might be influenced by the reduced accessibility of antenatal classes and MCHC services during the COVID-19 pandemic.

### Recommendations

In view of our findings, we propose six areas which the government and hospitals should address to further promote breastfeeding in Hong Kong.

First, staff training in private hospitals should be strengthened. Written notice of breastfeeding policies should be well-communicated to all staff (BFHI Step 1), while standard training should be mandated for staff (BFHI Step 2), especially for nurses and midwives in private maternity wards [21].

Second, a postpartum home-based programme should be implemented, as recommended by other local studies [17, 45]. Although open-ended responses suggested that practical, comprehensive, extensive, and interactive antenatal classes were welcomed by the mothers, the evenly divided rating of participants suggested that the helpfulness of existing antenatal classes was controversial. Results from other high-income countries consistently suggest that the efficacy of antenatal classes is uncertain [46]. In addition, mothers in Hong Kong may find it difficult to leave home during the early postpartum period due to the overwhelming workload and the cultural practice of “doing-the-month” [17]. Hence, the practical challenges of breastfeeding should be addressed postnatally in a home setting.

Third, breastfeeding support in SCBUs and NICUs of public hospitals should be strengthened. SCBUs and NICUs of public hospitals should offer clear breastfeeding guidance and support milk expression, bottle-feeding, and breastfeeding as appropriate.

Fourth, MCHC services should be continued during pandemics and be further promoted on discharge. The provision of information regarding breastfeeding support on discharge (BFHI Step 10) was associated with continued breastfeeding both locally and internationally [21, 26, 28]. While most public and private hospitals in Hong Kong self-reported that they provided such support, open-ended responses indicated that some participants were unaware of such services [5], suggesting a communication gap which should be addressed through active promotion.

Fifth, the spouse and the grandparents should be included in breastfeeding education and antenatal classes. Since family members play a significant role in the choice and duration of EBF, it is important to cultivate their knowledge on breastfeeding through family-centred breastfeeding education [47, 48].

Sixth, flexible work-from-home arrangements should be provided to parents following the end of existing parental leave. Descriptive statistics of this study indicated that the return to work from maternity leave is a common reason for EBF cessation among participants, while other studies indicated that parents’ return to work was significantly associated with early weaning [39, 45, 49]. Yet, the recent extension of statutory maternity leave to 14 weeks in Hong Kong still falls short of the six-month target, while statutory paternity leave lasts for 5 days only [4, 50]. With the experience of large-scale work-from-home arrangements during COVID-19, similar arrangements could be considered as an alternative to extending existing parental leave.

## Conclusions

This study identified the positive factors associated with six-month PBF in Hong Kong women, namely giving birth in a public hospital and breastfeeding support from family and friends. Furthermore, the COVID-19 pandemic in Hong Kong had an overall positive impact on six-month PBF. Further studies should investigate the impact of hospital practices and the COVID-19 pandemic on breastfeeding behaviours.

## Supplementary Information

**Additional file 1.** The original questionnaire (in English and Chinese)

## Data Availability

The datasets used and analysed during this study are available from the corresponding author on reasonable request.

## Abbreviations

BFHI: Baby-Friendly Hospital Initiative
BFHIHKA: Baby Friendly Hospital Initiative Hong Kong Association
CI: Confidence interval
EBF: Exclusive breastfeeding
HKBFMA: Hong Kong Breastfeeding Mothers’ Association
IBCLC: International Board Certified Lactation Consultant
LLLHK: La Leche League Hong Kong
MCHC: Maternal and child health centre
NICU: Neonatal intensive care unit.
OR: Odds ratio
PBF: Predominant breastfeeding.
SE: Standard error
SES: Socioeconomic status
SCBU: Special care baby unit
WHO: World Health Organization

## References

[1] Tarrant M. Submission to the legislative council panel on food safety and environmental hygiene and panel on health services on the Hong Kong code of marketing and quality of formula Milk and related products and food products for infants and young children. HK: School of Nursing, The University of Hong Kong; 2012. No.: CB(2)237/12-13(02).

[2] Hong Kong Code of Marketing of Formula Milk and Related Products, and Food Products for Infants & Young Children (HK) [cited 2022 Mar 2]; Available from: https://www.hkcode.gov.hk/en/the-hk-code-summary.html.

[3] Discrimination Legislation (Miscellaneous Amendments) Ordinance 2020 (HK).

[4] The Employment (Amendment) Ordinance 2020 (HK) [cited 2022 Mar 2]; Available from: URL: https://www.info.gov.hk/gia/general/202012/04/P2020120200260.htm.

[5] Baby Friendly Hospital Initiative Hong Kong Association (2020). World breastfeeding week (WBW) 1–7 august 2020.

[6] Lee SY. The 2018 National Survey on Fertility and Family Health and Welfare. KR: Institute for Health and Social Affairs; 2018. No.: 2018–37.

[7] Family Health Service. Breastfeeding survey 2019. HK: Department of Health; 2019. Available from: URL: https://www.fhs.gov.hk/english/archive/files/reports/BF_survey_2019.pdf.

[8] Inano H, Kameya M, Sasano K, Matsumura K, Tsuchida A, Hamazaki K (2021). Factors influencing exclusive breastfeeding rates until 6 months postpartum: the Japan environment and Children’s study. Sci Rep.

[9] Ministry of Health and Welfare. 母乳哺育國內現況 [Current breastfeeding situation in Taiwan]. https://www.hpa.gov.tw/Pages/Detail.aspx?nodeid=506&pid=463. Accessed 27 June 2021. Chinese.

[10] Office of the Surgeon General (2011). Barriers to breastfeeding in the United States.

[11] News.gov.hk. Work from home plan advised. https://www.news.gov.hk/eng/2020/07/20200727/20200727_180031_822.html. Accessed 27 June 2021.

[12] Family Health Service. Family Health Service – Temporary closure of 5 Maternal and Child Health Centres (MCHCs) and re-arrangement of service. https://www.fhs.gov.hk/english/news/. Accessed 27 June 2021.

[13] Hui PW, Ma G, Seto MTY, Cheung KW (2021). Effect of COVID-19 on delivery plans and postnatal depression scores of pregnant women. Hong Kong Med J.

[14] Leung H, Ip J, Chan A, Ngai P, Poon L (2021). How has COVID-19 impacted obstetrics?. Hong Kong J Gynaecol Obstet Midwifery.

[15] Latorre G, Martinelli D, Guida P, Masi E, De Benedictis R, Maggio L (2021). Impact of COVID-19 pandemic lockdown on exclusive breastfeeding in non-infected mothers. Int Breastfeed J.

[16] World Health Organization. Breastfeeding and replacement feeding practices in the context of mother-to-child transmission of HIV: an assessment tool for research: World Health Organization; 2001.

[17] Tarrant M, Dodgson JE, Wu KM (2014). Factors contributing to early breast-feeding cessation among Chinese mothers: an exploratory study. Midwifery..

[18] Baby Friendly Hospital Initiative Hong Kong Association. Myths on breastfeeding_FAQ. https://www.babyfriendly.org.hk/en/myths-on-breastfeeding_faq-2/. Accessed 27 June 2021.

[19] Family Health Service (2016). Survey on mothers’ breastfeeding experience in public places.

[20] Elo S, Kyngäs H (2008). The qualitative content analysis process. J Adv Nurs.

[21] World Health Organization. United Nations Children’s fund. Implementation guidance: protecting, promoting, and supporting breastfeeding in facilities providing maternity and newborn services: the revised baby-friendly hospital initiative. World Health Organization. 2018, p 12; Available from: URL: https://apps.who.int/iris/bitstream/handle/10665/272943/9789241513807-eng.pdf?ua=1.

[22] Gatti L (2008). Maternal perceptions of insufficient milk supply in breastfeeding. J Nurs Scholarsh.

[23] Hauck YL, Fenwick J, Dhaliwal SS, Butt J (2011). A Western Australian survey of breastfeeding initiation, prevalence and early cessation patterns. Matern Child Health J.

[24] Galipeau R, Baillot A, Trottier A, Lemire L (2018). Effectiveness of interventions on breastfeeding self-efficacy and perceived insufficient milk supply: a systematic review and meta-analysis. Matern Child Nutr..

[25] Tarrant M, Wu KM, Fong DY, Lee IL, Wong EM, Sham A (2011). Impact of baby-friendly hospital practices on breastfeeding in Hong Kong. Birth.

[26] Lok KYW, Chow CLY, Fan HSL, Chan VHS, Tarrant M (2020). Exposure to baby-friendly hospital practices and mothers’ achievement of their planned duration of breastfeeding. BMC Pregnancy Childbirth.

[27] Cascone D, Tomassoni D, Napolitano F, Di Giuseppe G. Evaluation of knowledge, attitudes, and practices about exclusive breastfeeding among women in Italy. Int J Environ Res Public Health. 2019;16(12). 10.3390/ijerph16122118. 10.3390/ijerph16122118 PMC6617343 31207988

[28] Pérez-Escamilla R, Martinez JL, Segura-Pérez S. Impact of the baby-friendly hospital initiative on breastfeeding and child health outcomes: a systematic review. Matern Child Nutr 2016;12(3):402–417. 10.1111/mcn.12294. 10.1111/mcn.12294 PMC6860129 26924775

[29] Oakley L, Benova L, Macleod D, Lynch CA, Campbell OMR. Early breastfeeding practices: descriptive analysis of recent demographic and health surveys. Matern Child Nutr 2018;14(2):e12535. 10.1111/mcn.12535. 10.1111/mcn.12535 PMC5900960 29034551

[30] World Health Organization. United Nations Children’s fund. Capture the moment - early initiation of breastfeeding: the best start for every newborn. World health Organization. 2018.

[31] Jaafar SH, Ho JJ, Lee KS. Rooming-in for new mother and infant versus separate care for increasing the duration of breastfeeding. Cochrane Database Syst Rev. 2016(8):Cd006641. 10.1002/14651858.CD006641.pub3. 10.1002/14651858.CD006641.pub3 PMC9168801 27562563

[32] Bastug A, Hanifehnezhad A, Tayman C, Ozkul A, Ozbay O, Kazancioglu S (2020). Virolactia in an asymptomatic mother with COVID-19. Breastfeed Med.

[33] Our World in Data. Italy: Coronavirus pandemic country profile. https://ourworldindata.org/coronavirus/country/italy. Accessed 27 June 2021.

[34] Remuzzi A, Remuzzi G (2020). COVID-19 and Italy: what next?. Lancet.

[35] Nolan M. Educators’ experience of facilitating antenatal education online. IJBPE. 2021.

[36] Kong SK, Lee DT (2004). Factors influencing decision to breastfeed. J Adv Nurs.

[37] Lok KYW, Bai DL, Tarrant M (2017). Family members’ infant feeding preferences, maternal breastfeeding exposures and exclusive breastfeeding intentions. Midwifery.

[38] Bai DL, Fong DY, Lok KY, Tarrant M (2016). Relationship between the infant feeding preferences of Chinese mothers’ immediate social network and early breastfeeding cessation. J Hum Lact.

[39] Flacking R, Dykes F, Ewald U (2010). The influence of fathers’ socioeconomic status and paternity leave on breastfeeding duration: a population-based cohort study. Scand J Public Health.

[40] Tarrant M, Dodgson JE, Tsang FS (2002). Initiating and sustaining breastfeeding in Hong Kong: contextual influences on new mothers’ experiences. Nurs Health Sci.

[41] Zhang K, Tang L, Wang H, Qiu L, Binns CW, Lee AH (2015). Why do mothers of young infants choose to formula feed in China? Perceptions of mothers and hospital staff. Int J Environ Res Public Health.

[42] Ku CM, Chow SK (2010). Factors influencing the practice of exclusive breastfeeding among Hong Kong Chinese women: a questionnaire survey. J Clin Nurs.

[43] Peregrino AB, Watt RG, Heilmann A, Jivraj S (2018). Breastfeeding practices in the United Kingdom: is the neighbourhood context important?. Matern Child Nutr..

[44] Census and Statistics Department. 2016 Population By-census. https://www.censtatd.gov.hk/en/scode459.html. Accessed 27 June 2021.

[45] Tarrant M, Fong DY, Wu KM, Lee IL, Wong EM, Sham A (2010). Breastfeeding and weaning practices among Hong Kong mothers: a prospective study. BMC Pregnancy Childbirth..

[46] Lumbiganon P, Martis R, Laopaiboon M, Festin MR, Ho JJ, Hakimi M (2016). Antenatal breastfeeding education for increasing breastfeeding duration. Cochrane Database Syst Rev.

[47] Su M, Ouyang YQ (2016). Father’s role in breastfeeding promotion: lessons from a quasi-experimental trial in China. Breastfeed Med.

[48] Susin LR, Giugliani ER (2008). Inclusion of fathers in an intervention to promote breastfeeding: impact on breastfeeding rates. J Hum Lact.

[49] Stuebe AM, Horton BJ, Chetwynd E, Watkins S, Grewen K, Meltzer-Brody S (2014). Prevalence and risk factors for early, undesired weaning attributed to lactation dysfunction. J Women's Health.

[50] The Employment Ordinance, Cap. 57 (HK).

