# Supplementary material for: A mixed-methods study on the association of six-month predominant breastfeeding with socioecological factors and COVID-19 among experienced breastfeeding women in Hong Kong
Source: Int Breastfeed J. 2022 May 21;17:40. doi: 10.1186/s13006-022-00484-7 (PMC9123774; doi:10.1186/s13006-022-00484-7)
Supplement: Supplementary file 1 — Additional file 1. The original questionnaire (in English and Chinese) [file 13006_2022_484_MOESM1_ESM.pdf]

Knowledge and Practice of Breastfeeding Amongst Mothers in Hong Kong Survey

Section 1: Personal information

1. Your age
   - ▢ 20 or below
   - ▢ 21 - 30
   - ▢ 31 - 40
   - ▢ 41 or above

2. Your educational attainment
   - ▢ No schooling / pre-primary
   - ▢ Primary
   - ▢ Lower secondary (Form 1 to form 3)
   - ▢ Upper secondary (Form 4 to form 7)
   - ▢ Post secondary: Diploma / certificate
   - ▢ Post secondary: Sub-degree course
   - ▢ Post secondary: Degree course

3. Your occupation
   - ▢ Managers and administrators
   - ▢ Professionals
   - ▢ Associate professionals
   - ▢ Clerical support workers
   - ▢ Service and sales workers
   - ▢ Craft and related workers
   - ▢ Plant and machine operators and assemblers
   - ▢ Elementary occupations
   - ▢ Housewife / homemaker [Skip to Q5]
   - ▢ Retired [Skip to Q5]
   - ▢ Between jobs [Skip to Q5]
   - ▢ Others (please specify)： ___________________

4. Your current mode of employment is:
   - ▢ Full time
   - ▢ Part time
   - ▢ On maternity leave

5. Your monthly total household income is: HKD ____________

6. Your residential property's saleable area is: _____________ square feet

7. Your household size is: _________ persons

Section 2: Breastfeeding situation

8. Age of your youngest child
   [Dropdown list]: Under 1 month, 1 month, 2 months, 3 months, 4 months, 5 months, 6 months, 7 months, 8 months, 9 months, 10 months, 11 months, 1 year, 2 years, 3 years, 4 years, 5 years, 6 years, 7 years, 8 years, 9 years, 10 years, 11 years, 12 years, 13 years, 14 years, 15 years, 16 years, 17 years, 18 years, above 18 years

9. This child was born in:
   - ▢ Public hospital
   - ▢ Private hospital

10. Feeding approach of this child during hospital stay after birth was:
    - ▢ Exclusive breastfeeding (not giving the infant any foods or beverages other than breast milk)
    - ▢ Mixed feeding (breast milk in combination with formula milk / milk powder / solid foods)
    - ▢ Only formula milk / milk powder
    - ▢ Not sure
    - ▢ Others (Please specify):_________

11. Has your child been discharged from the hospital?
    - ▢ Yes, already discharged
    - ▢ No, not yet discharged

12. Since when was this child breastfed?
    - ▢ Never breastfed [Skip to Q14]
    - ▢ The __ month after birth (enter “0” if breastfed since birth)
    - ▢ Not sure

13. When did this child stop being breastfed?
    - ▢ The __ month after birth
    - ▢ Still being breastfed
    - ▢ Not sure

14. Since when was this child given formula milk / milk powder?
   - ▢ Never given formula milk / milk powder [Skip to Q16]
   - ▢ The __ month after birth (enter “0” if given formula milk / milk powder since birth)
   - ▢ Not sure

15. When did this child stop drinking formula milk / milk powder?
   - ▢ The __ month after birth
   - ▢ Still drinking formula milk / milk powder
   - ▢ Not sure

16. Since when was this child given solid foods?
   - ▢ Never given solid foods
   - ▢ The __ month after birth (enter “0” if given solid foods since birth)
   - ▢ Not sure

17. After hospital discharge, has this child been hospitalised for treatment due to neonatal jaundice?
   - ▢ Yes
   - ▢ No [Skip to Q20]

18. The feeding approach of this child for the duration of hospital stay due to neonatal jaundice was:
   - ▢ Exclusive breastfeeding
   - ▢ Mixed feeding
   - ▢ Formula milk / milk powder only
   - ▢ Others (Please specify): ________

19. The feeding approach of this child after discharge from hospital upon completion of treatment was:
   - ▢ Exclusive breastfeeding
   - ▢ Mixed feeding
   - ▢ Formula milk / milk powder only
   - ▢ Others (Please specify): ________

20. If your child has been exclusively breastfed, why did you **exclusively** breastfeed your child? (You may choose more than one answer)
    - ▢ Out of maternal love
    - ▢ Out of a sense of responsibility
    - ▢ Out of peer pressure
    - ▢ Exclusive breastfeeding is healthier for the child
    - ▢ Exclusive breastfeeding is healthier for the mother
    - ▢ Exclusive breastfeeding is cheaper
    - ▢ Exclusive breastfeeding is simpler / more convenient
    - ▢ My family members strongly support / encourage so
    - ▢ My friends strongly support / encourage so
    - ▢ Others (Please specify): ________
    - ▢ My child has not been exclusively breastfed

21. If your child is not being **exclusively** breastfed currently, why did you stop exclusive breastfeeding or choose not to breastfeed **exclusively**? (You may choose more than one answer)
    - ▢ Natural weaning
    - ▢ Low breast milk supply
    - ▢ Busy life
    - ▢ Returned to work after maternity leave ended
    - ▢ Not supported by the workplace
    - ▢ Not supported by colleagues
    - ▢ Not supported by family members
    - ▢ Latching issues
    - ▢ Breast pain
    - ▢ Physical illness
    - ▢ Pregnancy
    - ▢ Peer pressure
    - ▢ Others (Please specify): ________
    - ▢ My child is still being exclusively breastfed currently

Section 3: Breastfeeding knowledge

22. ”The contents of formula milk is the same as breast milk.” Is this statement correct or incorrect?
    - ▢ Correct
    - ▢ Incorrect
    - ▢ I don’t know

23. ”It is normal to experience pain during breastfeeding.” Is this statement correct or incorrect?
    - ▢ Correct
    - ▢ Incorrect
    - ▢ I don’t know

24. ”The size and shape of the nipple will affect breastfeeding.” Is this statement correct or incorrect?
    - ▢ Correct
    - ▢ Incorrect
    - ▢ I don’t know

25. ”Breastfeeding should be regular, once every four hours.” Is this statement correct or incorrect?
    - ▢ Correct
    - ▢ Incorrect
    - ▢ I don’t know

26. Have you ever come across promotional materials regarding breastfeeding or infant / child diet from the Department of Health?
    - ▢ Yes
    - ▢ No [Skip to Q28]

27. Your source(s) of information was/were: (You may choose more than one answer)

- ▢ Electronic media (including television, radio)
- ▢ Poster / pamphlets (for example in clinics, hospitals etc.)
- ▢ Print media (including newspapers, magazines)
- ▢ Health facilities / Healthcare professionals
- ▢ The Internet (including YouTube, websites)
- ▢ Public transport
- ▢ Others (Please specify): ________
- ▢ Do not remember

Section 4: Breastfeeding support from family and friends

28. ”My spouse is supportive of me breastfeeding my child.”
    - ▢ Strongly disagree
    - ▢ Disagree
    - ▢ Neutral
    - ▢ Agree
    - ▢ Strongly agree
    - ▢ Not applicable

29. ”My parents are supportive of me breastfeeding my child.”
    - ▢ Strongly disagree
    - ▢ Disagree
    - ▢ Neutral
    - ▢ Agree
    - ▢ Strongly agree
    - ▢ Not applicable

30. ”My friends are supportive of me breastfeeding my child.”
    - ▢ Strongly disagree
    - ▢ Disagree
    - ▢ Neutral
    - ▢ Agree
    - ▢ Strongly agree
    - ▢ Not applicable

Section 5: Breastfeeding support from hospitals and organisations

31. Have you attended antenatal talks / classes?
    - ▢ Yes
    - ▢ No [Skip to Q35]

32. Around how much time was spent on teaching breastfeeding?
    - ▢ 15 minutes or less
    - ▢ 15-30 minutes
    - ▢ 30-60 minutes
    - ▢ 1-2 hours
    - ▢ 2-3 hours
    - ▢ 3 hours or more

33. Do you agree that the antenatal talk / class you attended was enough in guiding you to breastfeed your child?
    - ▢ Strongly disagree
    - ▢ Disagree
    - ▢ Neutral
    - ▢ Agree
    - ▢ Strongly agree

34. Referring to the previous questions, the reason is: ____________

35. How helpful were the maternity ward nurses' / midwives' teachings on breast pumping?
    - ▢ Not at all helpful
    - ▢ Slightly helpful
    - ▢ Quite helpful
    - ▢ Very helpful

36. Have you encountered the following difficulties in maternity wards? (You may choose more than one answer)
    ▢ Need to supplement with other milk sources due to low breast milk supply
    ▢ Nurses were too busy to offer effective breastfeeding assistance
    ▢ Midwives were too busy to offer effective breastfeeding assistance
    ▢ Unable to initiate breastfeeding within 30 minutes after delivery / becoming responsive
    ▢ Latching issues
    ▢ Did not encounter any aforementioned difficulties
    ▢ Others (Please specify): ________

37. After hospital discharge, have you ever sought lactation consultation due to breastfeeding difficulties?
    ▢ Yes
    ▢ No [Skip to Q56]

38. Have you seeked lactation consultation from the following organisations? (You may choose more than one answer)
    ▢ Maternal and Child Health Centre [Please answer Q39]
    ▢ Breastfeeding support organisations (for example Hong Kong Breastfeeding Mothers' Association, breastfeeding support groups) [Please answer Q42 and Q43]
    ▢ Breastfeeding clinic in the hospital that your child was born in [Please answer Q44 and Q45]
    ▢ International Board Certified Lactation Consultant [Please answer Q46 and Q47]
    ▢ Private doctor [Please answer Q48 and Q49]
    ▢ Private nurse [Please answer Q50 and Q51]
    ▢ Confinement nanny [Please answer Q52 and Q53]
    ▢ Milk enhancing worker [Please answer Q54 and Q55]
    ▢ Others (please specify): ____________

39. Did you find the Maternal and Child Health Centre helpful?
    ▢ Not at all helpful [Please answer Q40]
    ▢ Slightly helpful
    ▢ Quite helpful
    ▢ Very helpful

40. Referring to the previous question, the reason(s) is/are: (you may choose more than one answer)
    ▢ Nurses were too busy to offer effective breastfeeding assistance
    ▢ Unclear explanation
    ▢ Fail to perform at home
    ▢ Others (please specify): ________________

41. [If “Maternal and Child Health Centre” was not selected in Q38]
    The reason(s) you did not seek lactation consultation from Maternal and Child Health Centres is/are: (you may choose more than one answer)
    ▢ The Maternal and Child Health Centre was too far away
    ▢ Scheduling conflict
    ▢ It is difficult to leave home for the Maternal and Child Health Centre after birth
    ▢ I have already obtained lactation consultation from elsewhere
    ▢ Others (please specify): ________

42. Did you find the breastfeeding support organisation helpful?
    ▢ Not at all helpful
    ▢ Slightly helpful
    ▢ Quite helpful
    ▢ Very helpful

43. Referring to the previous questions, the reason is: ____________

44. Did you find the breastfeeding clinic in the hospital that your child was born in helpful?
    ▢ Not at all helpful
    ▢ Slightly helpful
    ▢ Quite helpful
    ▢ Very helpful

45. Referring to the previous questions, the reason is: ____________

46. Did you find the International Board Certified Lactation Consultant helpful?
    ▢ Not at all helpful
    ▢ Slightly helpful
    ▢ Quite helpful
    ▢ Very helpful

47. Referring to the previous questions, the reason is: ____________

48. Did you find the private doctor helpful?
- ▢ Not at all helpful
- ▢ Slightly helpful
- ▢ Quite helpful
- ▢ Very helpful

49. Referring to the previous questions, the reason is: ____________

50. Did you find the private nurse helpful?
- ▢ Not at all helpful
- ▢ Slightly helpful
- ▢ Quite helpful
- ▢ Very helpful

51. Referring to the previous questions, the reason is: ____________

52. Did you find the confinement nanny helpful?
- ▢ Not at all helpful
- ▢ Slightly helpful
- ▢ Quite helpful
- ▢ Very helpful

53. Referring to the previous questions, the reason is: ____________

54. Did you find the milk enhancing worker helpful?
- ▢ Not at all helpful
- ▢ Slightly helpful
- ▢ Quite helpful
- ▢ Very helpful

55. Referring to the previous questions, the reason is: ____________

Section 6: Breastfeeding support from public premises and the workplace

56. Have you ever breastfed in public?
    - ▢ Yes
    - ▢ No [Skip to Q59]

57. Have you ever experienced discrimination in public premises because of breastfeeding?
    - ▢ Yes
    - ▢ No [Skip to Q59]

58. Select the unpleasant experiences that you have encountered. (You may choose more than one answer)
    - ▢ Being stared at
    - ▢ Being photographed or filmed
    - ▢ Being requested to stop breastfeeding
    - ▢ Being requested to leave the premise
    - ▢ Being verbally harassed
    - ▢ Being physically harassed
    - ▢ Others (please specify): ___________

[Q59 to Q63 were displayed for participants who answered Q4]

59. Does your workplace adopt any "Breastfeeding-friendly Workplace" measures?
    - ▢ Yes
    - ▢ No
    - ▢ I don't know

60. Is the support for breastfeeding at your workplace sufficient?
    - ▢ Very insufficient
    - ▢ Insufficient
    - ▢ Sufficient
    - ▢ Very sufficient

61. Is the support for breast milk expression at your workplace sufficient?
- ▢ Very insufficient
- ▢ Insufficient
- ▢ Sufficient
- ▢ Very sufficient

62. Which of the following breastfeeding-friendly measures are offered at your workplace? (You may choose more than one answer)
- ▢ Allowing lactation breaks for breast milk expression
- ▢ A space with privacy
- ▢ A chair
- ▢ A table
- ▢ An electrical socket
- ▢ A refrigerator for storing breast milk
- ▢ None of the above
- ▢ Others (please specify): ______________

63. Have you ever experienced discrimination at your workplace because of breastfeeding?
- ▢ Yes
- ▢ No

Section 7: Political support for breastfeeding

64. According to the Employment (Amendment) Ordinance 2020, statutory maternity leave is extended from 10 weeks to 14 weeks. In your opinion, how helpful is this amendment in supporting breastfeeding among employed mothers?
    - ▢ Not at all helpful
    - ▢ Slightly helpful
    - ▢ Quite helpful
    - ▢ Very helpful
    - ▢ Not sure

65. The Discrimination Legislation (Miscellaneous Amendments) Ordinance 2020 protects breastfeeding women from direct or indirect discrimination. In your opinion, how helpful is this amendment in supporting breastfeeding among employed mothers?
    - ▢ Not at all helpful
    - ▢ Slightly helpful
    - ▢ Quite helpful
    - ▢ Very helpful
    - ▢ Not sure

66. Do you agree with establishing breast milk banks to serve mothers who are unable to breastfeed?
    - ▢ Strongly disagree
    - ▢ Disagree
    - ▢ Neutral
    - ▢ Agree
    - ▢ Strongly agree

Section 8: Impact of COVID-19 pandemic on breastfeeding

67. [Displayed for participants who answered Q4]
    Has your workplace ever adopted any work-from-home policies during the pandemic?
    - ▢ Yes
    - ▢ No

68. Have you breastfed or considered breastfeeding during the pandemic?
    - ▢ Yes
    - ▢ No [End of survey]

69. On the whole, has the COVID-19 pandemic discouraged/encouraged you to breastfeed?
    Discouraged → No effect → Encouraged (0-10 points slider)

70. Referring to the previous question, the reason(s) is/are: (You may choose more than one answer)
    - ▢ I can work from home [Displayed for participants who answered Q4]
    - ▢ My work schedule became more flexible [Displayed for participants who answered Q4]
    - ▢ I leave home less during the pandemic
    - ▢ My family members can better support breastfeeding at home
    - ▢ Breastfeeding can improve infants' immunity
    - ▢ It is easier to seek help in regards to breastfeeding
    - ▢ It is more difficult to seek help in regards to breastfeeding
    - ▢ I want to minimise physical contact with my children, so as to reduce their chance of being infected
    - ▢ I want to avoid breastfeeding/expressing breast milk at the workplace, so as to reduce my children's chance of being infected [Displayed for participants who answered Q4]
    - ▢ I want to avoid breastfeeding/expressing breast milk in public, so as to reduce my children's chance of being infected
    - ▢ Others (please specify): _____________

香港母親對母乳餵哺的認識和實踐
問卷調查

第一部分：個人資料

1. 您的年齡
   - ▢ 20 歲或以下
   - ▢ 21 - 30 歲
   - ▢ 31 - 40 歲
   - ▢ 41 歲或以上

2. 您的教育程度
   - ▢ 未受教育 / 學前教育
   - ▢ 小學
   - ▢ 初中（中一至中三）
   - ▢ 高中/預科（中四至中七）
   - ▢ 專上教育：文憑 / 證書
   - ▢ 專上教育：副學位
   - ▢ 專上教育：學位

3. 您的職業
   - ▢ 經理及行政級人員
   - ▢ 專業人員
   - ▢ 輔助專業人員
   - ▢ 文書支援人員
   - ▢ 服務工作及銷售人員
   - ▢ 工藝及有關人員
   - ▢ 機台及機器操作員及裝配員
   - ▢ 非技術工人
   - ▢ 家庭主婦 / 料理家務【請跳至第 5 題】
   - ▢ 退休【請跳至第 5 題】
   - ▢ 待業【請跳至第 5 題】
   - ▢ 其他（請註明）：___________________

4. 您現在的工作形式是:
    - ▢ 全職
    - ▢ 兼職
    - ▢ 正在放產假

5. 您每月的家庭總收入為:港幣____________

6. 您的家居實用面積為:____________平方呎

7. 您的家庭成員數目為:____________人

第二部分：母乳餵哺情況

8. 最年幼孩子的年齡
   [Dropdown list]: 1月以下，1月，2月，3月，4月，5月，6月，7月，8月，9月，10月，11月，1歲， 2歲， 3歲， 4歲， 5歲， 6歲， 7歲， 8歲， 9歲， 10歲， 11歲， 12歲， 13歲， 14歲， 15歲， 16歲， 17歲， 18歲， 18歲以上

9. 該孩子出生於：
   - ▢ 公立醫院
   - ▢ 私家醫院

10. 孩子出生後於住院期間的餵哺方式為：
    - ▢ 純母乳餵哺（即不給予嬰兒母乳以外任何食物或飲料）
    - ▢ 混合母乳餵哺（配合配方奶 / 奶粉 / 固體食物）
    - ▢ 純配方奶 / 奶粉
    - ▢ 不清楚
    - ▢ 其他（請註明）：________

11. 孩子現在是否已經出院？
    - ▢ 是，已出院
    - ▢ 否，仍未出院

12. 孩子何時開始以母乳餵哺？
    - ▢ 從沒有以母乳餵哺【請跳至第 14 題】
    - ▢ 出生後第___個月（若出生時已開始母乳餵哺，請輸入「0」）
    - ▢ 不清楚

13. 孩子何時停止以母乳餵哺？
    - ▢ 出生後第___個月
    - ▢ 現在仍母乳餵哺中
    - ▢ 不清楚

14. 孩子何時開始飲用配方奶 / 奶粉？
    - ▢ 從沒有飲用配方奶 / 奶粉【請跳至第 16 題】
    - ▢ 出生後第___個月（若出生時已開始飲用配方奶 / 奶粉，請輸入「0」）
    - ▢ 不清楚

15. 孩子何時停止飲用配方奶 / 奶粉？
 - ▢ 出生後第___個月
 - ▢ 現在仍飲用配方奶 / 奶粉
 - ▢ 不清楚

16. 孩子何時開始進食固體食物？
 - ▢ 從沒有進食固體食物
 - ▢ 出生後第___個月（若出生時已開始進食固體食物，請輸入「0」）
 - ▢ 不清楚

17. 出院後，該孩子曾否因新生兒黃疸而需再度入院治療？
 - ▢ 有
 - ▢ 否【請跳至第 20 題】

18. 該孩子因新生兒黃疸入院治療期間的餵哺方式為：
 - ▢ 純母乳餵哺
 - ▢ 混合母乳餵哺
 - ▢ 純配方奶 / 奶粉
 - ▢ 其他（請註明）：________

19. 該孩子療程完畢出院後的餵哺方式為：
 - ▢ 純母乳餵哺
 - ▢ 混合母乳餵哺
 - ▢ 純配方奶 / 奶粉
 - ▢ 其他（請註明）：________

20. 若您曾以<u>純</u>母乳餵哺，你為何以<u>純</u>母乳餵哺？（可選多項）
 - ▢ 出於母愛
 - ▢ 出於責任感
 - ▢ 出於群眾壓力
 - ▢ 純母乳餵哺對孩子身體更健康
 - ▢ 純母乳餵哺對母親身體更健康
 - ▢ 純母乳餵哺較為便宜
 - ▢ 純母乳餵哺較為簡單 / 方便
 - ▢ 家人極力支持 / 鼓勵
 - ▢ 朋友極力支持 / 鼓勵
 - ▢ 其他（請註明）：____________
 - ▢ 沒有以純母乳餵哺

21. 若您現在沒有以純母乳餵哺，請問您為何停止 / 不以純母乳餵哺？（可選多項）

- ▢ 自然離乳
- ▢ 母乳不足
- ▢ 生活忙碌
- ▢ 產假結束後重返工作
- ▢ 工作場所不支持
- ▢ 同事不支持
- ▢ 家人不支持
- ▢ 吸啜問題
- ▢ 乳房疼痛
- ▢ 身體不適
- ▢ 懷孕
- ▢ 群眾壓力
- ▢ 其他（請註明）：____________
- ▢ 現在仍以純母乳餵哺

第三部分：對母乳餵哺的認知

22.「配方奶的成分與母乳相同。」這句話是正確還是錯誤的？
- ▢ 正確
- ▢ 錯誤
- ▢ 不知道

23.「餵哺母乳時感到疼痛是正常的。」這句話是正確還是錯誤的？
- ▢ 正確
- ▢ 錯誤
- ▢ 不知道

24.「乳頭的大小和形狀會影響餵哺。」這句話是正確還是錯誤的？
- ▢ 正確
- ▢ 錯誤
- ▢ 不知道

25.「餵哺母乳應該有規律，每四小時餵哺一次。」這句話是正確還是錯誤的？
- ▢ 正確
- ▢ 錯誤
- ▢ 不知道

26. 您曾否接觸衛生署有關母乳餵哺或嬰幼兒飲食的推廣訊息？
- ▢ 有
- ▢ 否【請跳至第 28 題】

27. 您接觸有關訊息的途徑是：（可選多項）
- ▢ 電子傳媒（包括電視、電台）
- ▢ 海報/宣傳單張（例如在診所、醫院等等）
- ▢ 文字傳媒（包括報章、雜誌）
- ▢ 醫護機構/醫護人員
- ▢ 互聯網（包括 YouTube、網頁）
- ▢ 公共交通工具
- ▢ 其他（請註明）：____________
- ▢ 記不起

第四部分：家人和朋友對母乳餵哺的支援

28.「我的伴侶支持我餵哺母乳。」
- ▢ 非常不同意
- ▢ 不同意
- ▢ 中立
- ▢ 同意
- ▢ 非常同意
- ▢ 不適用

29.「我的父母支持我餵哺母乳。」
- ▢ 非常不同意
- ▢ 不同意
- ▢ 中立
- ▢ 同意
- ▢ 非常同意
- ▢ 不適用

30.「我的朋友支持我餵哺母乳。」
- ▢ 非常不同意
- ▢ 不同意
- ▢ 中立
- ▢ 同意
- ▢ 非常同意
- ▢ 不適用

第五部分：醫院和機構對母乳餵哺的支援

31. 您曾否參加產前講座 / 課程？
    - ▢ 有
    - ▢ 否【請跳至第 35 題】

32. 大約多少時間是用於教授母乳餵哺？
    - ▢ 15 分鐘
    - ▢ 15 - 30 分鐘
    - ▢ 30 分鐘-1 小時
    - ▢ 1 小時
    - ▢ 2 小時
    - ▢ 3 小時或以上

33. 您同意所參加的產前講座 / 課程足以引導您進行母乳餵哺嗎？
    - ▢ 非常不同意
    - ▢ 不同意
    - ▢ 中立
    - ▢ 同意
    - ▢ 非常同意

34. 承上題，原因為：____________

35. 產科病房的護士 / 助產士有關泵奶的教導對您有多少幫助？
    - ▢ 沒有幫助
    - ▢ 有一點幫助
    - ▢ 很有幫助
    - ▢ 非常有幫助

36. 您在產科病房裏曾否遇到以下困難？（可選多項）
    - ▢ 護士過於忙碌而未能有效提供對母乳餵哺的協助
    - ▢ 助產士過於忙碌而未能有效提供對母乳餵哺的協助
    - ▢ 未能於產後 / 清醒後 30 分鐘內開始餵哺母乳
    - ▢ 吸啜問題
    - ▢ 醫生說不夠奶要加奶
    - ▢ 沒有遇到以上困難
    - ▢ 其他（請註明）：_________

37. 您出院後因母乳餵哺困難，而尋求哺乳諮詢？
  - ▢ 有
  - ▢ 否【請跳至第 56 題】

38. 您曾否向以下機構尋求哺乳諮詢？（可選多於一項）
  - ▢ 母嬰健康院【請回答第 39 題】
  - ▢ 母乳餵哺支援組織（如母乳育嬰協會、母親互助小組等）【請回答第 42 和 43 題】
  - ▢ 孩子出世的醫院的母乳餵哺診所【請回答第 44 和 45 題】
  - ▢ 國際認証哺乳顧問【請回答第 46 和 47 題】
  - ▢ 私家醫生【請回答第 48 和 49 題】
  - ▢ 私家護士【請回答第 50 和 51 題】
  - ▢ 陪月員【請回答第 52 和 53 題】
  - ▢ 催乳師【請回答第 54 和 55 題】
  - ▢ 其他（請註明）：____________

39. 您認為母嬰健康院有幫助嗎？
  - ▢ 沒有幫助【請回答第 40 題】
  - ▢ 有一點幫助
  - ▢ 很有幫助
  - ▢ 非常有幫助

40. 承上題，原因是：（可選多項）
  - ▢ 護士過於忙碌而未能有效提供對母乳餵哺的協助
  - ▢ 講解不清晰
  - ▢ 回到家後做不到
  - ▢ 其他（請註明）：________________

41. 【若第 38 題沒有選擇「母嬰健康院」】
    您沒有向母嬰健康院尋求哺乳諮詢的原因是：（可選多項）
  - ▢ 母嬰健康院距離遙遠
  - ▢ 時間衝突
  - ▢ 產後難以外出前往母嬰健康院
  - ▢ 已從其他途徑滿足哺乳諮詢的需要
  - ▢ 其他（請註明）：________

42. 您認為母乳餵哺支援組織有幫助嗎？

- ▢ 沒有幫助
- ▢ 有一點幫助
- ▢ 很有幫助
- ▢ 非常有幫助

43. 承上題，原因為：_____________

44. 您認為孩子出世的醫院的母乳餵哺診所有幫助嗎？

- ▢ 沒有幫助
- ▢ 有一點幫助
- ▢ 很有幫助
- ▢ 非常有幫助

45. 承上題，原因為：_____________

46. 您認為國際認証哺乳顧問有幫助嗎？

- ▢ 沒有幫助
- ▢ 有一點幫助
- ▢ 很有幫助
- ▢ 非常有幫助

47. 承上題，原因為：_____________

48. 您認為私家醫生有幫助嗎？

- ▢ 沒有幫助
- ▢ 有一點幫助
- ▢ 很有幫助
- ▢ 非常有幫助

49. 承上題，原因為：_____________

50. 您認為私家護士有幫助嗎？

- ▢ 沒有幫助
- ▢ 有一點幫助
- ▢ 很有幫助
- ▢ 非常有幫助

51. 承上題, 原因為:_____________

52. 您認為陪月員有幫助嗎？
  - ▢ 沒有幫助
  - ▢ 有一點幫助
  - ▢ 很有幫助
  - ▢ 非常有幫助

53. 承上題, 原因為:_____________

54. 您認為催乳師有幫助嗎？
  - ▢ 沒有幫助
  - ▢ 有一點幫助
  - ▢ 很有幫助
  - ▢ 非常有幫助

55. 承上題, 原因為:_____________

<u>第六部分：公眾場所及公司對母乳餵哺的支援</u>

56. 您曾否於公眾場所進行母乳餵哺？
    - ▢ 有
    - ▢ 否【請跳至第 59 題】

57. 您曾否於公眾場所因母乳餵哺而受到歧視？
    - ▢ 有
    - ▢ 否【請跳至第 59 題】

58. 請選出您遇到的不愉快經歷（可選多項）
    - ▢ 被他人注視
    - ▢ 被拍照或錄影
    - ▢ 被要求停止餵哺母乳
    - ▢ 被要求離開場所
    - ▢ 被言語上騷擾 / 恐嚇
    - ▢ 被肢體上騷擾 / 恐嚇
    - ▢ 其他（請註明）：__________

【第 59 至 63 題只供已回答第 4 題之參與者作答】

59. 您所在的公司有實施「母乳餵哺友善工作間」嗎？
    - ▢ 有
    - ▢ 否
    - ▢ 不知道

60. 您所在的公司對母乳餵哺的支援足夠嗎？
    - ▢ 非常不足夠
    - ▢ 不足夠
    - ▢ 足夠
    - ▢ 非常足夠

61. 您所在的公司對泵奶的支援足夠嗎？
    - ▢ 非常不足夠
    - ▢ 不足夠
    - ▢ 足夠
    - ▢ 非常足夠

62. 在以下支援母乳餵哺的措施當中，您所在的公司提供哪些？(可選多項)

- ▢ 容許僱員利用授乳時段擠母乳
- ▢ 具備私隱的空間
- ▢ 座椅
- ▢ 桌子
- ▢ 電插座
- ▢ 可存放母乳的雪櫃
- ▢ 一項也沒有
- ▢ 其他(請註明)：_______

63. 您曾否於公司因母乳餵哺而受到歧視？

- ▢ 有
- ▢ 否

第七部分：政策上對母乳餵哺的支援

64. 根據《2020年僱傭（修訂）條例》，法定產假由 10 周延長至 14 周。您認為此修訂對鼓勵在職母親進行母乳餵哺有多大程度上的幫助？
    - ▢ 沒有幫助
    - ▢ 有一點幫助
    - ▢ 很有幫助
    - ▢ 非常有幫助
    - ▢ 不清楚

65. 《2020年歧視條例（雜項修訂）條例》明文保障餵哺母乳的婦女免受直接或間接歧視。您認為此修訂對鼓勵在職母親進行母乳餵哺有多大程度上的幫助？
    - ▢ 沒有幫助
    - ▢ 有一點幫助
    - ▢ 很有幫助
    - ▢ 非常有幫助
    - ▢ 不清楚

66. 您是否贊同成立母乳庫為不能餵哺母乳的母親服務？
    - ▢ 非常不贊同
    - ▢ 不贊同
    - ▢ 中立
    - ▢ 贊同
    - ▢ 非常贊同

第八部分：新冠疫情對母乳餵哺的影響

67.【只供已回答第 4 題之參與者作答】
您曾否於新冠疫情期間「在家工作」(work-from-home)？
- ▢ 有
- ▢ 否

68. 您在新冠疫情期間曾否(考慮)進行母乳餵哺？
- ▢ 有
- ▢ 否【全問卷完畢】

69. 新冠疫情整體上有否妨礙 / 鼓勵您進行母乳餵哺？
妨礙 → 沒有影響 → 鼓勵 (0-10 points slider)

70. 承上題，原因為：(可選多項)
- ▢ 我能在家工作【只供已回答第 4 題之參與者作答】
- ▢ 我的工作時間表更有彈性【只供已回答第 4 題之參與者作答】
- ▢ 我在疫情期間較少出門
- ▢ 我的家人更能在家支持母乳餵哺
- ▢ 母乳餵哺能增加嬰兒免疫力
- ▢ 更容易尋求母乳餵哺方面的幫助
- ▢ 更難以尋求母乳餵哺方面的幫助
- ▢ 我想盡量減少與孩子的身體接觸，以降低孩子的感染風險
- ▢ 我想盡量避免於工作場所餵哺母乳/泵奶，以降低孩子的感染風險【只供已回答第 4 題之參與者作答】
- ▢ 我想盡量避免於公共場所餵哺母乳/泵奶，以降低孩子的感染風險
- ▢ 其他(請註明)：____________
